# Machine Learning Models for Multiparametric Glioma Grading With Quantitative Result Interpretations

**DOI:** 10.3389/fnins.2018.01046

**Published:** 2019-01-11

**Authors:** Xiuying Wang, Dingqian Wang, Zhigang Yao, Bowen Xin, Bao Wang, Chuanjin Lan, Yejun Qin, Shangchen Xu, Dazhong He, Yingchao Liu

**Affiliations:** ^1^School of Information Technologies, The University of Sydney, Sydney, NSW, Australia; ^2^Department of Pathology, Provincial Hospital Affiliated to Shandong University, Jinan, China; ^3^School of Medicine, Shandong University, Jinan, China; ^4^Department of Neurosurgery, Provincial Hospital Affiliated to Shandong University, Jinan, China; ^5^School of Information and Communication Engineering, Beijing University of Posts and Telecommunications, Beijing, China

**Keywords:** glioma grading, machine learning, morphological features, support vector machine, digital pathology images

## Abstract

Gliomas are the most common primary malignant brain tumors in adults. Accurate grading is crucial as therapeutic strategies are often disparate for different grades and may influence patient prognosis. This study aims to provide an automated glioma grading platform on the basis of machine learning models. In this paper, we investigate contributions of multi-parameters from multimodal data including imaging parameters or features from the Whole Slide images (WSI) and the proliferation marker Ki-67 for automated brain tumor grading. For each WSI, we extract both visual parameters such as morphology parameters and sub-visual parameters including first-order and second-order features. On the basis of machine learning models, our platform classifies gliomas into grades II, III, and IV. Furthermore, we quantitatively interpret and reveal the important parameters contributing to grading with the Local Interpretable Model-Agnostic Explanations (LIME) algorithm. The quantitative analysis and explanation may assist clinicians to better understand the disease and accordingly to choose optimal treatments for improving clinical outcomes. The performance of our grading model was evaluated with cross-validation, which randomly divided the patients into non-overlapping training and testing sets and repeatedly validated the model on the different testing sets. The primary results indicated that this modular platform approach achieved the highest grading accuracy of 0.90 ± 0.04 with support vector machine (SVM) algorithm, with grading accuracies of 0.91 ± 0.08, 0.90 ± 0.08, and 0.90 ± 0.07 for grade II, III, and IV gliomas, respectively.

## Introduction

Gliomas are the most common primary malignant brain tumors in adults, accounting for 30% of all primary central nervous system (CNS) tumors and 80% of all malignant brain tumors (Goodenberger and Jenkins, 2012). According to their histological characteristics, such as cellularity, pleomorphism, nuclear atypia, necrosis, and endothelial proliferation, gliomas can be classified into WHO grades I–IV (Louis et al., 2007). Correctly differentiating tumor grades is critical because they are widely used to predict patient outcomes and determine the use of adjuvant therapy protocols including aggressive radiotherapy and concomitant chemotherapy (Hsu et al., 1997). A precise prediction of the prognosis depends on an accurate pathology diagnosis. Compared to glioblastoma (GBM), low-grade gliomas (LGGs; WHO II) have greater chemotherapy sensitivity and a better post-therapy prognosis (Cillekens et al., 2000; Cavaliere et al., 2005; Wang and Jiang, 2013).

Considered as one of the “gold standards” and conventionally used by pathologists for tumor grading, haematoxylin and eosin (H&E) stained images provide specific histological characteristics and patterns to differentiate gliomas grades (Rubin et al., 2008). The parameters extracted by clinicians from WSIs mostly focused on the cellular level, including the number and size of nuclei, which reflects the proliferation and differentiation of the nuclei per unit area and heterogeneity of different levels of the tumor cells. For instance, compared to grade II gliomas, grade III, and grade IV both exhibit microvascular proliferation (MVP), indicating the presence of proliferation of enlarged blood vessels in the tissue (Burger et al., 2002). Grade IV gliomas can be further distinguished from grade III by examining H&E stained images for the presence of highly pleomorphic cells with hyperchromatic, irregular nuclei and brisk mitotic activity. Grade IV gliomas are often more mitotically active, necrosis prone, and generally associated with neovascularity, infiltration of surrounding tissue and a rapid postoperative progression (Louis et al., 2016). However, the conventional grading procedure based on H&E pathology images is often subjective and operator-dependent and of low reproducibility due to the inter-observer variability.

Apart from H&E stained images, immunocytochemical staining with Ki-67 antibodies has been widely accepted as an alternative reference for assessing the proliferative potential in tumor cells. Strictly associated with cell proliferation, Ki-67 nuclear antigen is present during all active phases of the cell cycle (Gap 1, Synthesis, and Gap 2 phases of the cell cycle) and mitosis but absent in resting (quiescent) cells (Gap 0) (Bruno and Darzynkiewicz, 1992). The proliferative index (PI), determined by Ki-67 immunohistochemistry (IHC), correlates well with the histological malignancy grade of gliomas (Skjulsvik et al., 2014). PI values may assist in differentiating grade II from III or grade II from IV; however, due to the overlapping PI values of grades III and IV, PI itself would not be considered as sufficient evidence to adequately determine these two malignancy grades (Skjulsvik et al., 2014).

Computer-assisted grading systems are capable of mining a large number of quantitative features from digital pathology slide images to sort out the most important patterns for grading. This ability provides the opportunity for better quantitative modeling of the disease appearance and hence possibly improves the prediction accuracy of tumor grading. In addition, it also provides a more reproducible, less labor-intensive and more efficient mechanism than manual grading by pathologists. However, the compelling opportunities offered by big digital pathology data come with optimal computational algorithm challenges (Madabhushi and Lee, 2016). For example, image analysis and computer-assisted detection models inadequately address the data density in high-resolution digitized whole slide images (WSI). Mousavi et al. (2015) proposed to distinguish LGGs from GBMs using image features extracted from regions of interest (ROIs) in WSIs. However, the manual selection of ROI in this method may introduce a potential risk of inter-observer variance. Kong et al. (2009) tiled the WSI into non-overlapping pieces and analyzed each image tile to grade LGGs from GBMs. While fully processing all the image tiles, the method might be less computationally efficient, in particular, for high-resolution H&E stained images. To improve computational efficiency of the automated prognosis of neuroblastoma from H&E images, Sertel et al. (2009) proposed a multiresolution approach to extract local binary pattern and texture features of different scales.

While the above mentioned automated methods process and analyze WSIs for grading, the method proposed by Ertosun and Rubin (2015) classified LGGs and high-grade glioma (HGGs) on the basis of automated segmentation and analysis of cell nuclei and morphological features (Kong et al., 2013) but neglected the tumor patterns important for manual grading. To systematically compare the performance of classic machine learning models on grading, Huang and Lee (2009) implemented three machine learning models, Bayesian, k-nearest neighbors (KNN) and support vector machine (SVM), and concluded that KNN and SVM both achieved the highest accuracy. Apart from conventional machine learning, Reza and Iftekharuddin, 2016 proposed a deep learning-based grading system, characterizing each tile type with convolutional neural network (CNN). However, these machine learning and deep learning models are considered as “black boxes” without transparent interpretation of either the models themselves or the grading results.

To improve reproducibility and avoid inter-operator variance in conventional manual grading, in this study, we design and implement a platform for automated grading gliomas into grades II, III, and IV from digital pathology images. In our approach, discriminative visual, sub-visual and IHC parameters are identified, and a reliable machine learning model is selected. With the machine learning based models, we integrate information from both histological morphology images and proliferation biomarkers into a single unified framework to predict the glioma grade in 116 patients,which surpass the current clinical paradigm for patients diagnosed with glioma. Our platform provides interpretation on the grading outcomes to disclose the contributions of multiparametric features to individual cases and presents an alternative for objective, accurate, and interpretable prediction of glioma grading in the clinic.

## Materials and Methods

### Material and Dataset

The dataset used in this study involves 146 cases of glioma grading from grades II to IV: 49 grade II, 45 grade III, and 52 grade IV images. All cases were from Shandong Provincial Hospital affiliated to Shandong University, and the pathology diagnoses of the cases were based on WHO standards (Louis et al., 2007). Paraffin-embedded samples were cut into 3 μm thick sections and stained with H&E stain. All H&E images in this study were obtained from WSIs scanned by a Leica SCN400 slide scanner (Leica Biosystems, Nussloch, Germany) with multiresolution varying from 20× to 40×. Ki-67 immunohistochemical staining was prepared using an automated staining instrument (Ventana, Benchmark Ultra). As defined in Eq. 1, Ki-67 PI is the percentage of the number of immunoreactive tumor cells in relation to the total number of cells. At least 1000 tumor cells or alternatively, three high-power fields (HPFs) were examined by two independent experienced observers. The mean of Ki-67 PI is the average of the values calculated by different observers.

(1) $$\mathrm{PI}=\frac{\mathrm{positive}\;\mathrm{cells}}{\mathrm{total}\;\mathrm{cells}}*100$$

### Automated Interpretable Glioma Grading With Machine Learning Models

**FIGURE 1:**
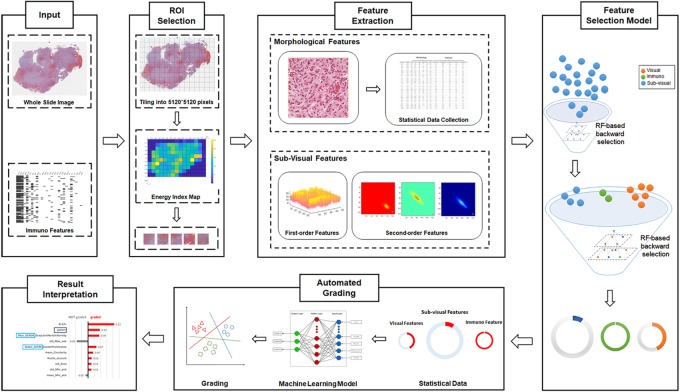
Schematic flowchart of the automated grading framework. We first automatically selected the representative regions of interest (ROIs) from the H&E images. Based on these ROIs, we extracted and selected important visual, sub-visual, and immunohistochemical features. We established automated machine learning models with these features for glioma grading. The grading results output from the model were further explained with the LIME algorithm.

As illustrated in Figure 1, our automated grading framework is composed of five major components including automated ROI identification, feature extraction, important feature selection, automated grading, and result interpretation.

### Automated ROI Identification

Our proposed computational method automatically identifies the ROIs that reflect cell proliferation and cellular density based on the number of nuclei in the regions. H&E images were first partitioned as tiles, each with a resolution of 5120^∗^5120 pixels to enable the process of high-resolution imaging (Sertel et al., 2009; Mobadersany et al., 2018). Then, we detected nuclei using watershed nuclei detection algorithm in each tile (Al-Kofahi et al., 2010; Kumar et al., 2017) and based on the density of the detected nuclei, the five tiles with the highest densities of nuclear were identified as the ROIs.

### Multi-Parameter Extraction and Important Parameter Selection

From the identified ROIs, we extracted multi-parameters including visual features and sub-visual features as the inputs for automated grading. The visual features, including seven nuclear morphological features, five nuclear staining features, and nuclei clusters or patterns, were extracted to reflect a basis for observation when pathologists make diagnostic decisions (Kong et al., 2013). For instance, nuclear morphological characteristics such as shape, size, and circularity that reflect cellular atypia have been commonly used by pathologists to distinguish different grades of glioma.

In addition to the visual features, sub-visual features also contribute to accurate glioma grading. The sub-visual features are the computerized high-throughput intensity and texture image features that have been proved to have diagnostic, predictive, and prognostic power, although these features are somehow beyond human perception capabilities (Thawani et al., 2017). In our method, for instance, intensity features describe the first-order statistical information of the image intensity distribution, while the second-order grey-level co-occurrence matrix (GLCM) features capture both statistical intensity and relationship between neighborhood pixels, revealing information such as homogeneity, contrast and entropy (Selvarajah and Kodituwakku, 2011). As illustrated in Figure 1, we used a random forest (RF)-based feature selection method combined with backward feature elimination to choose the most representative and informative features (Saeys et al., 2007).

### Machine Learning Model for Automated Grading

**FIGURE 2:**
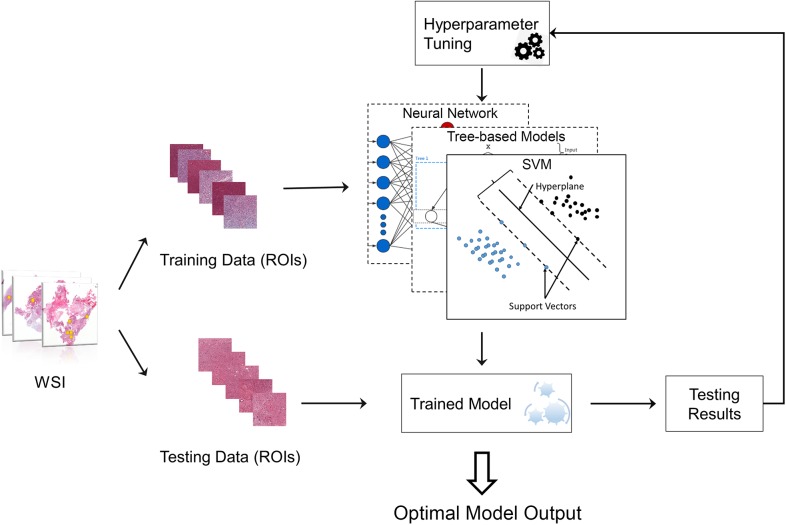
Machine learning model with hyperparameter tuning. The original data were randomly separated into training data and testing data. Training data were used to train the machine learning model, while testing data were used to evaluate the model performance. The hyperparameters of the model can be continuously tuned with information acquired from testing results until the optimal model is obtained.

To select the best machine learning model for grading, we compared the performance of four types of classic machine learning models including RF (Liaw and Wiener, 2002), gradient boosting decision tree (GBDT) (Friedman, 2002), SVM (Furey et al., 2000) and neural network (Hagan et al., 1996) (NN) (Zacharaki et al., 2009; Kothari et al., 2013). To achieve the best performance for each grading model, the hyperparameters of the model need to be optimized, for example, the tree number in RF model. The framework for automatically tuning the hyperparameters is provided in Figure 2.

### Validation and Evaluation

116 patients out of 146 was used to train the model while the other 30 patients was used for further validate the model. The 116 patients were randomly divided into training datasets (75%) and testing datasets (25%) to test performance and validate the models (Larsen and Goutte, 1999). The corresponding training set was used to train the classifier, and the test set was used to verify the model performance. This procedure was repeated 30 times by changing random states while maintaining the same train-test split ratio. Apart from accuracy, other measurements including precision, recall, F1 score and confusion matrix were calculated to more completely validate grading performance. In addition, extra 30 cases were used as validation dataset with 30 times of cross-validation to further test the performance of our proposed model.

## Results

### Multi-Parameter Extraction and Selection

**FIGURE 3:**
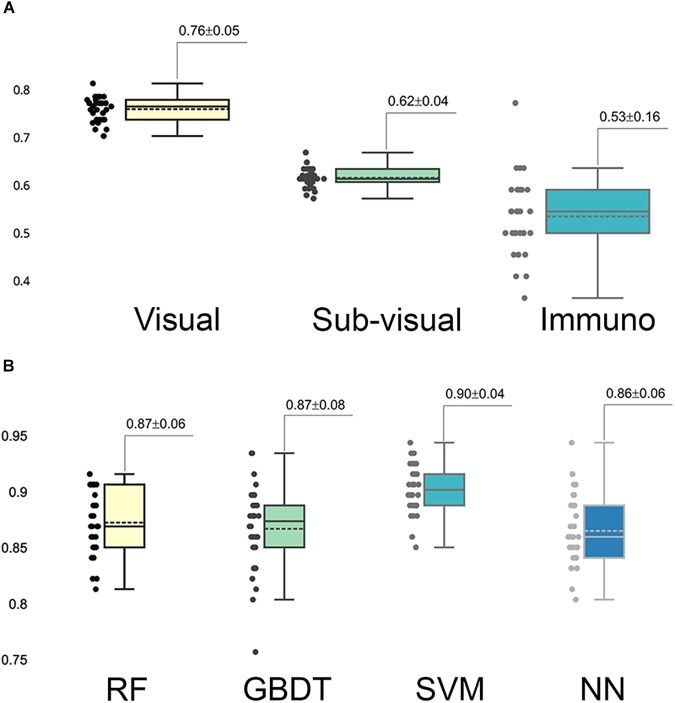
Predictive capability of selected important features and models. **(A)** Predictive accuracy of different categories of selected important features. In each category, features were assessed for accuracy separately. **(B)** Accuracy of different grading models. Each model was assessed with 30 times cross-validation.

In the multi-parameter extraction phase, we extracted 24 visual parameters and 171 sub-visual parameters. Since the large size of the sub-visual features may suppress important morphological features, we performed the feature selection process on the sub-visual features twice to eventually choose 15 features from this category. The predictive performance of the selected 11 visual features, 15 sub-visual features and one IHC characteristic – Ki-67 in this study – were assessed with separate RF models. The box-plot in Figure 3A shows that visual features had the highest predictive power with 0.76 accuracy, while the accuracy for sub-visual features and Ki-67 only reached 0.62 and 0.53, respectively.

### Automated Grading Performance With Different Models

We fed these selected important parameters into the four machine learning models RF, GBDT, SVM, and NN to sort out the best grading model. Based on cross-validation, SVM achieved the most accurate and stable grading performance, with the accuracy of 0.90 ± 0.04, while the other three models had lower but nearly the same performance with accuracy stabilized at approximately 0.87 (Figure 3B). Table 1 shows the results of other measurements including F1, precision and recall, among which the SVM model maintained the best performance.

**Table 1:** F1, accuracy, precision, and recall for different machine learning models.

Method	F1	Accuracy	Precision	Recall
Random forest	0.86 ± 0.07	0.87 ± 0.06	0.86 ± 0.07	0.87 ± 0.06
GBDT	0.86 ± 0.08	0.87 ± 0.07	0.86 ± 0.07	0.86 ± 0.07
Neural network	0.86 ± 0.05	0.86 ± 0.06	0.87 ± 0.06	0.87 ± 0.05
SVM	0.90 ± 0.07	0.90 ± 0.04	0.91 ± 0.04	0.91 ± 0.04


### Further Investigation of SVM Performance

**FIGURE 4:**
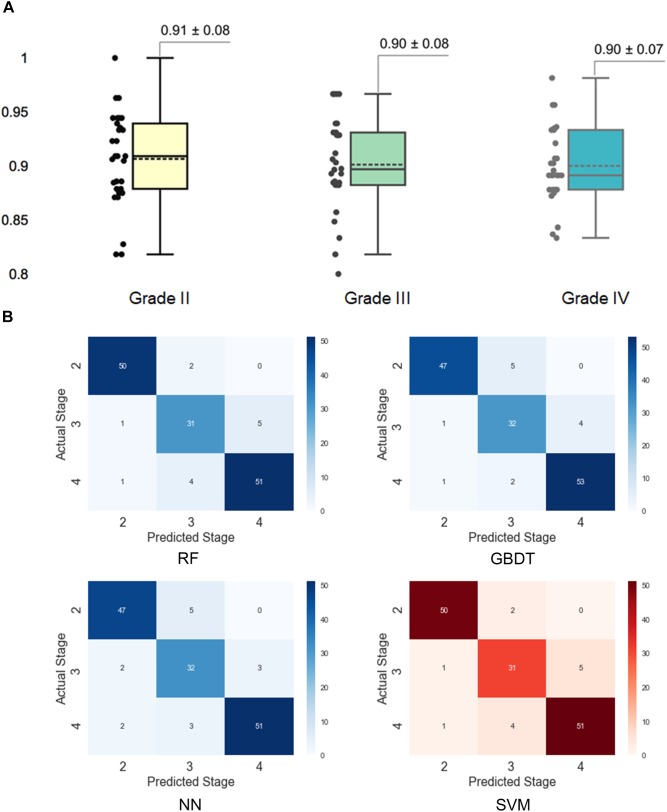
Assessment of grading results with the support vector machine (SVM) model. **(A)** Prediction accuracy for different histological grades in the SVM model. The accuracy for each grade was obtained by separating the results by grades in the 30 times cross-validation. **(B)** Confusion matrixes for different models. The confusion matrixes reveal the number of misclassified cases for each grade.

To better understand the performance of the SVM model for grading histologic grades of glioma II, III, and IV, we calculated a confusion matrix to uncover misclassification cases. As illustrated in Figure 4A, grade II had the highest accuracy (0.91) in the SVM model while the other two grades both had the accuracy of 0.90. As shown in the confusion matrix for the SVM model (Figure 4B), grade III gliomas may share common histological characteristics with both grade II and grade IV gliomas and thereby may lead to misclassification. In addition, we added 30 new patients of grade II to IV for the further validation. The validation performance achieved 0.88 ± 0.14 with 30 times cross-validation.

### Quantitative Grading Results Interpretation

The machine learning algorithm is usually complex and considered as a “black box” without explicit interpretation of the learning process or the outputs. To provide a better quantitative interpretable explanation for the grading results, we used Local Interpretable Model-Agnostic Explanations (LIME) algorithm (Ribeiro et al., 2016) to reveal the importance of features and their underlying contribution to the grading decision. In this section, we illustrated three case studies to interpret the automated grading results and the underlying reasoning for our SVM model with the LIME algorithm.

**FIGURE 5:**
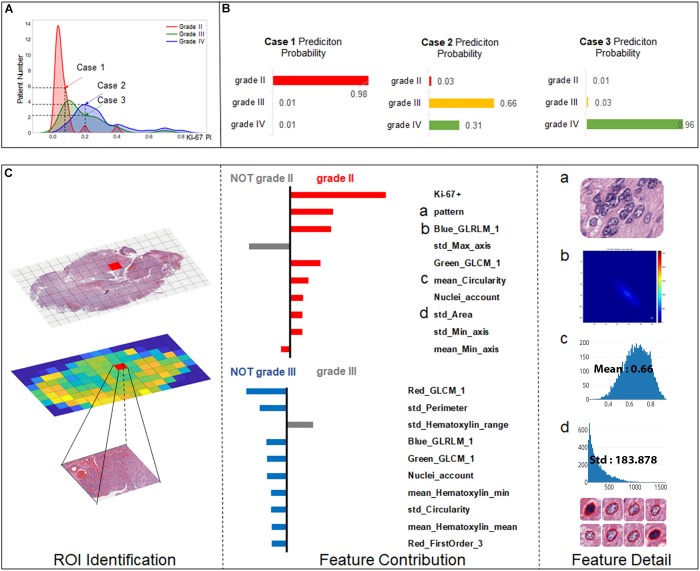
Grading result explanation of representative cases with the LIME algorithm for the SVM model (part 1) **(A)** Distribution of Ki-67 by different grades. Three selected cases had Ki-67 PI values of 0.08, 0.2, and 0.2. **(B)** Prediction probability for 3 cases. **(C)** Glioma Case 1 for grade II with a Ki-67 PI value of 0.08, and Ki-67 PI was the dominant feature.

As Ki-67 has been proved to be a clinically significant indicator for grading, we predicted the grading results by machine learning models while also taking the influence of Ki-67 into consideration. To highlight this strategy, we chose three cases to elucidate the workflow of SVM for precise grading. As shown in Figure 5A, Case 1 is a relatively easy case to classify, being grade II with low Ki-67 (0.05). However, Case 2 and Case 3 are more ambiguous, both with a value of 0.2 for Ki-67, but one is grade III while the other is grade IV. We established a result explainer with LIME algorithm for the SVM model and then calculated the feature contribution for each case.

Case 1 (Figure 5C) was correctly classified as grade II with a high probability. As Ki-67 has been clinically proved to have the discriminative capacity to distinguish between LGG and HGG, it is not surprising that Ki-67 has the dominant influence underlying this correct decision. Apart from Ki-67, visual features such as cell pattern and sub-visual features such as GLCM texture features also can correctly guide the grading. The result also shows that although some morphological features such as perimeter do not support the decision of grade II, they excluded grade III, which also contributed to accurate grading.

**FIGURE 6:**
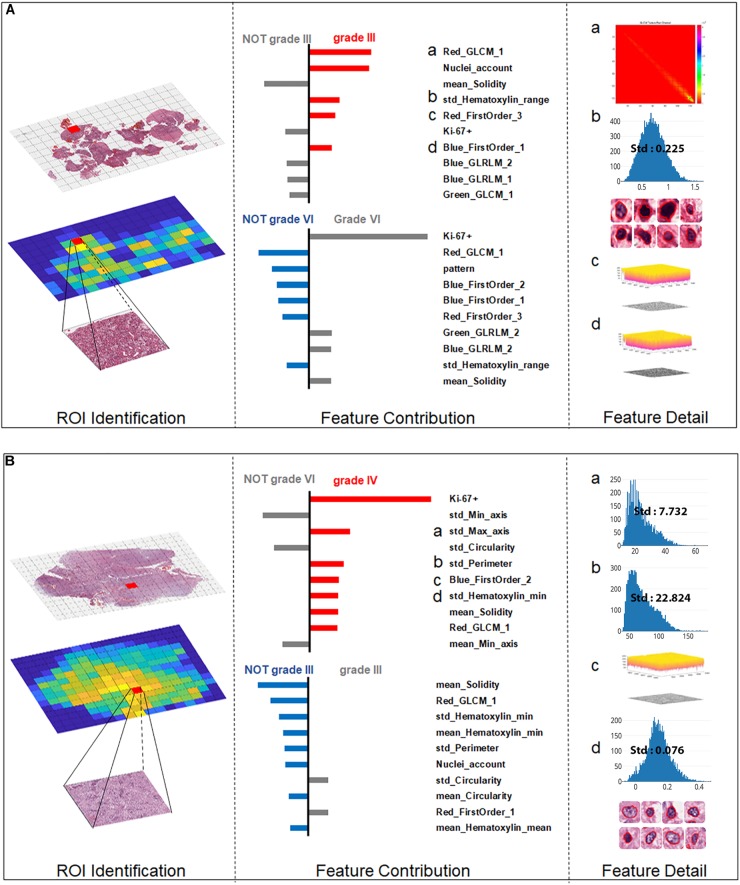
Grading result explanation of representative cases with the Local Interpretable Model-Agnostic Explanations (LIME) algorithm for the SVM model (part 2) **(A)** Glioma Case 2 for grade III with Ki-67 PI value of 0.2 while texture features and morphological nuclei count feature were the major contributors. **(B)** Glioma Case 3 for grade IV also with Ki-67 PI value of 0.2 accounting for the dominant contribution.

For the more ambiguous Case 2 (Figure 6A) and Case 3 (Figure 6B), with regard to Ki-67 itself, our classification model demonstrated its capability for an accurate decision. Case 2 (Figure 5B) was the most challenging case among these three cases because in this case, Ki-67 had only a negative influence on the grading decision; however, with the strong support of GLCM texture features and the morphological nuclei count feature, our grading system made a correct decision. In Case 3, Ki-67 was the major contributor to the correct decision, while the morphological features such as standard deviation of cells’ max axis and standard deviation of cells’ perimeter were the second and third contributors.

## Discussion

Given the well-recognized difficulties associated with the grading of gliomas, in this study, we developed an automated and interpretable grading of gliomas with a machine learning platform to provide an additional reference for diagnostic decision making and patient management and prognosis, which is more efficient, effective, and objective than currently used methods. Our major findings were that (1) our method achieved a high grading accuracy of 90% for classifying gliomas into grades II, III, and IV; and (2) more importantly, for the first time, an interpretable insight into the grading outputs was provided for the histology grading system with the “black-box” machine learning models.

Computerized image perception in tissue histology is much more difficult due to the complexity of the cell patterns and inherent structural associations between different tissue components. Accurate histopathological diagnosis determines patient management, treatment, and follow-up, so any method that results in more objective glioma grading may be of great value. In the current literature, automated grading either was based on texture patterns extracted from the selected ROIs (Mousavi et al., 2015) to capture the histological structures in the histological images or utilized morphological features extracted from segmented tumor cells (Sertel et al., 2009; Ertosun and Rubin, 2015). Our method achieved high accuracy through fully utilizing the selected informative visual and sub-visual features from H&E images combined with the Ki-67 PI value. To take into account the visual cues used by pathologists for manual grading, visual features such as texture patterns and cell morphological characteristics were extracted from selected ROIs. The sub-visual features that are beyond human perception capacities can help the precise grading through more comprehensive quantitative analyses. Furthermore, we innovatively integrated the IHC indicator Ki-67 PI into our grading platform, which contributed to boosting our grading accuracy with high discriminatory power.

Generally, the aim of this research was to objectify, standardize and quantify features that are already widely accepted as important by pathologists. Inter-observer discrepancies, which had long been a diagnostic problem in the past, could be partly overcome by employing the morphometric analysis used in the present study (Kinjo et al., 2008). The ability of the system to objectively identify regions of tumor may additionally complement the pathologist’s diagnosis and assist in tailored treatment. In this study, we attempted to automatically clarify the cellular composition and histopathological features of different grades of gliomas by utilizing morphometric- and immunohistochemical-based machine learning models. We assessed the importance of visual features, sub-visual features and Ki-67 separately, and the highest predictive power was achieved by visual features, achieving an accuracy of 0.76, which indicates that the histomorphology of glioma, such as nuclear morphological features and nuclear staining features and patterns play an important role in glioma grading.

The interpretability of the individuals’ grading results provided quantitative insights into the feature contributions of each individual case. Most of the grading systems based on machine learning models are considered “black boxes,” and it would be valuable for patient management if clinically trusted reasoning could be revealed. In our study, we used the LIME algorithm to provide an explanation for each individual grading result. Case 1 and Case 3 in Figures 5C, 6B endorse the important role that Ki-67 plays in grading decisions, which is in accordance with the clinical fact that Ki-67 PI is positively correlated with the histopathology grades (Faria et al., 2006). However, Ki-67 sometimes has a detrimental influence on the grading process, as shown in Case 2, because Ki-67 PI of grade III gliomas largely overlaps with that of grade IV (Shibata et al., 1988; Kayaselçuk et al., 2002; Johannessen and Torp, 2006; Skjulsvik et al., 2014). As a result, Ki-67 itself cannot be the only determinant of grading. It has been accepted that the Ki-67 PI value is 3.0 ± 2.1% in grade II gliomas, 11.8 ± 3.4% in grade III anaplastic gliomas and 15.8 ± 7.4% in grade IV GBMs (Johannessen and Torp, 2006). Furthermore, the Ki-67 PI values of grade III anaplastic glioma can overlap with values of grade II glioma at one end of the range and with those of GBM at the other in the diagnostic practice of pathologists. Apart from Ki-67, morphological features including the standard deviation of cells’ max axis and perimeter were also found to be a significant contributor to glioma grading because HGG often exhibits strong heterogeneity with irregular cell shapes (Shuangshoti et al., 2000; Louis et al., 2016). The precise morphological features extracted from the most aggressive regions help to investigate intratumoral histological heterogeneity for precise histopathology grading (Walker et al., 2001). The primary grading results still support the conclusion that the only sure way to determine the histopathological WHO grade remains the pathohistological evaluation of the H&E stained tumor sample (Stoyanov et al., 2017).

With three representative cases we could gain a deeper understanding of why misclassified grade III cases are often labeled as grade II, while grade IV cases could be wrongly labeled grade III as shown in the confusion matrix. Actually, the Ki-67 PI values of the GBM group can be as low as those for grade II tumors, indicating the limitation of Ki-67 values in the overlap region. In our work, we introduced a number of histological sub-visual features such as the intensity and GLCM texture features to distinguish these three grades of gliomas. These insights become easy once it is understood what the algorithm models are actually doing, which in turn leads to models that generalize much better results. In many applications of machine learning, users are asked to trust a model to help them make decisions. There has always been a focus on “trust” in any type of modeling methodology, but with machine learning, many people feel that the black-box approach taken with these methods is not trustworthy. Through this machine learning-based approach, we could use the LIME algorithm to explain individual predictions to the decision-maker (the pathologist), and that understanding of the model’s predictions can be an additional useful tool when deciding whether a model is trustworthy or not for the final diagnosis from a pathologist.

Our study did, however, have its limitations. First, grade I gliomas are not included in our studies. They are accurately considered benign in clinical practice, in that complete surgical excision is considered curative. Therefore, grade I gliomas are different from grade II-IV gliomas in biological behaviour. The results of our discrimination of grade III and grade IV are just reasonable preliminary results but leave much room for improvement. Considering that necrosis is one of the remarkable features of GBM, we plan to use cell necrosis as an input feature to further train models to distinguish grade III from grade IV GBM. Actually, morphometric data research has indicated that the cellularity of oligodendrogliomas type II was significantly higher than that of diffuse astrocytomas and that the conditional entropy of oligodendrogliomas type III was significantly lower than that of diffuse astrocytomas (Faria et al., 2006), so further stratification of LGG (grade II) will lead to tailored glioma management according to their different biological behaviour. Hence, future work will focus on improvements utilizing larger datasets, including multi-centre cases. In addition, there is still much room to improve the grading performance and generalization of the algorithm model.

## Conclusion

In conclusion, our approach provides an objective alternative for quantitative pathology research and for the implementation of morphological data in routine diagnostic practice. The machine learning model utilized multi-parameters including morphometric and sub-visual parameters as well as Ki-67 PI information to ensure high accuracy, efficiency and consistency in glioma grading. Interpretable grading platform has the potential to facilitate personalized medicine in the setting of malignant gliomas. With different important features identified for different patients, specific phenotypic tumor characteristics can be uncovered for optimal treatment selection. In addition, as our method is fully automated and quantitative with high reliability, it becomes easier for our platform to be introduced in routine clinical practice.

## Ethics Statement

Does the study presented in the manuscript involve human or animal subjects: Yes.

This study was carried out in accordance with the recommendations of 1964 Declaration of Helsinki and its later amendments. Written Informed consent was obtained from all patients, and this study was approved by the Ethics Committee of Provincial Hospital affiliated to Shandong University (LCYJ: No2017-035).

## Author Contributions

XW, YL, and SX designed the experiments. YL, CL, and YQ conducted H&E slide imaging. ZY and CL performed the immunohistochemical analysis. XW, DW, BW, BX, and DH designed and implemented the computational platform and statistical analyses. XW, DW, BX, ZY, and YL drafted the manuscript. XW and YL initiated and supervised the entire project.

## Conflict of Interest Statement

The authors declare that the research was conducted in the absence of any commercial or financial relationships that could be construed as a potential conflict of interest.
